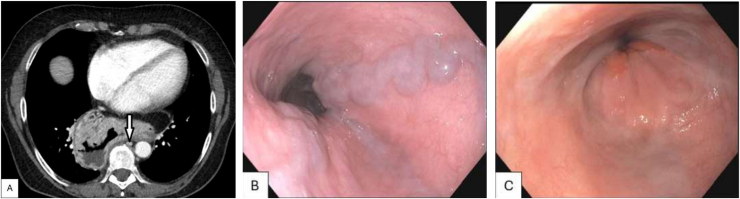# Downhill Danger: A Rare Case of Esophageal Varices From Hiatal Hernia-Induced Compression

**DOI:** 10.1016/j.gastha.2025.100777

**Published:** 2025-08-25

**Authors:** Miguel E. Gomez, Harmeet Malhi, Douglas Simonetto

**Affiliations:** 1 Division of Internal Medicine, Mayo Clinic, Rochester, Minnesota; 2 Division of Gastroenterology and Hepatology, Mayo Clinic, Rochester, Minnesota

This is a 63-year-old female presenting to clinic with worsening epigastric pressure and heartburn. She had been diagnosed on esophagogastroduodenoscopy (EGD) with a large hiatal hernia and esophageal varices at an outside hospital. EGD was repeated and she was found to have 2 large columns of isolated distal esophageal varices (Figure B). Hepatic venous gradient was normal. Liver biopsy reported normal lobular architecture with minimal steatosis and no fibrosis. Additionally, pathological inflammation, Mallory-Denk Bodies, and ballooning degeneration were absent in the liver biopsy. Trichrome stain was normal. Superior vena cava obstruction was ruled out with computed tomography venogram, which showed a large hiatal hernia with extrinsic compression of the azygos vein (Figure A, demonstrated by arrow). Patient underwent robotic assisted laparoscopic paraoesophageal hernia repair with partial fundoplication with thoracic surgery for symptom management. Clinic visit after surgery showed 50% reduction in reflux and pressure-like symptoms. Repeat EGD showed significantly decreased size of varices (Figure C). Therefore, leading hypothesis on cause of her isolated esophageal varices was mass effect from her large hiatal hernia resulting in azygous vein compression.

**Figure:**